# Quality assurance in severe sepsis: an individualised audit/feedback system results in substantial improvements at a UK teaching hospital

**DOI:** 10.1186/cc12438

**Published:** 2013-03-19

**Authors:** M Simmonds, E Blyth, W Robson, M Chikhani

**Affiliations:** 1 Nottingham University Hospitals NHS Trust, Nottingham, UK

## Introduction

Following our study of severe sepsis care across three centres [1], we aimed to introduce a rapid feedback mechanism into our rolling audit programme. Whilst previous audits raised awareness of severe sepsis, only whole organisation performance was reported and no feedback was given to individual clinicians. It is recognised that such feedback loops can improve clinical practice [2].

## Methods

**Figure 1:**
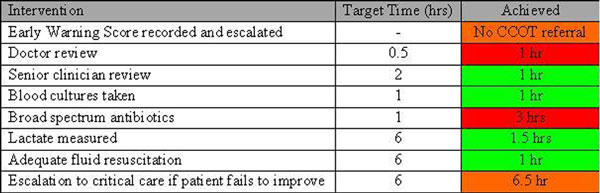
**Example report**.

Patients admitted to critical care (58 beds, four units) with a primary admission diagnosis of infection were screened for severe sepsis. Pre-ICU care was then audited against the Surviving Sepsis Guidelines [3]. Time zero is defined as when criteria for severe sepsis were first met. An individualised traffic-light report was then generated and emailed to the patient's consultant and other stakeholders involved in care (Figure 1). We aimed to report cases within 7 days of critical care admission. A cumulative report is generated monthly to track organisation-wide performance.

## Results

**Figure 2:**
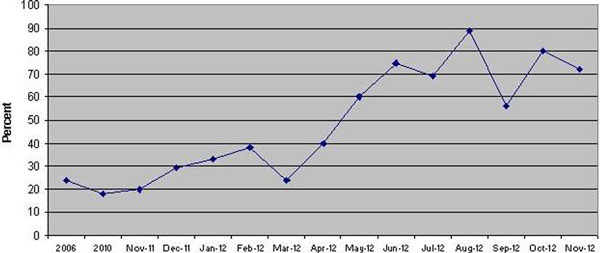
**Compliance with pre-ICU resuscitation bundle**.

Since November 2011, 153 cases of severe sepsis have been audited and reported back to clinicians. Compliance with antibiotics in <1 hour has risen from 35 to 75% and compliance with the pre-ICU elements of the resuscitation bundle has risen from 20 to 70% (Figure 2). Feedback from clinicians has been encouraging as our reports highlight both positive and negative examples of practice.

## Conclusion

Individualised feedback on sepsis care has led to substantial improvements in guideline compliance. This concept could be translated to other time-dependent patient pathways.
